# A Semi-Supervised Method for Drug-Target Interaction Prediction with Consistency in Networks

**DOI:** 10.1371/journal.pone.0062975

**Published:** 2013-05-07

**Authors:** Hailin Chen, Zuping Zhang

**Affiliations:** 1 School of Information Science and Engineering, Central South University, Changsha, China; 2 Department of Computer Science and Technology, Hunan University of Humanities, Science and Technology, Loudi, China; Koç University, Turkey

## Abstract

Computational prediction of interactions between drugs and their target proteins is of great importance for drug discovery and design. The difficulties of developing computational methods for the prediction of such potential interactions lie in the rarity of known drug-protein interactions and no experimentally verified negative drug-target interaction sample. Furthermore, target proteins need also to be predicted for some new drugs without any known target interaction information. In this paper, a semi-supervised learning method NetCBP is presented to address this problem by using labeled and unlabeled interaction information. Assuming coherent interactions between the drugs ranked by their relevance to a query drug, and the target proteins ranked by their relevance to the hidden target proteins of the query drug, we formulate a learning framework maximizing the rank coherence with respect to the known drug-target interactions. When applied to four classes of important drug-target interaction networks, our method improves previous methods in terms of cross-validation and some strongly predicted interactions are confirmed by the publicly accessible drug target databases, which indicates the usefulness of our method. Finally, a comprehensive prediction of drug–target interactions enables us to suggest many new potential drug–target interactions for further studies.

## Introduction

Drug discovery is an expensive and time-consuming process. Each year, only around 20 new drugs known as New Molecular Entities (NMEs) are approved by US Food and Drug Administration (FDA) (http://www.fda.gov/Drugs/DevelopmentApprovalProcess/HowDrugsareDevelopedandApproved/DrugandBiologicApprovalReports/default.htm). Meanwhile, the updated database of SuperTarget [1] curates 196 000 drug compounds (including approved drugs). As the paradigm of ’one gene, one drug, one disease’ has been challenged, the concept of polypharmacology has been proposed for those drugs acting on multiple targets rather than one target [2], [3]. Such polypharmacological features enable us to find their new uses, namely drug repositioning [4], and to understand drug side effects. Therefore, the identification of drug-target interactions is critical in drug discovery.

As experimental approaches for potential drug-target interactions remain challenging [5], [6], computational prediction methods are needed to solve this problem. To date, a variety of *in silico* methods have been developed to predict interactions between drugs and their targets.

The conventional computational methods can be categorized into ligand-based approach [7], receptor-based approach [8] and literature text mining approach [9]. However, all the three techniques have their limitations. The performance of the ligand-based approaches depends on the number of known ligands for a target protein of interest. The receptor-based approaches like docking cannot be applied to targets whose three-dimensional (3D) structures are unknown. The text mining approaches suffer from the problem of redundancy in the compound/gene names in the literature [9].

More recently, several statistical methods have been developed to infer potential drug-target interactions under the assumption that similar ligands are likely to interact with similar proteins. The prediction is conducted by integrating some biological information, such as drug chemical structures, target protein sequences and currently known compound-protein interactions. Yamanishi et al. [10] first characterized four classes of drug-target interaction networks and introduced a supervised method to infer unknown drug–target interactions by integrating chemical space and genomic space into a unified space called ‘pharmacological space’. Bleakley and Yamanishi [11] used bipartite local models (BLM) to infer unknown drug-target interactions. Yamanishi et al. [12] further investigated the relationship between the chemical space, the pharmacological space and the topology of drug-target interaction networks, and developed a method to predict unknown drug-target interactions from chemical, genomic and pharmacological data on a large scale. Gönen [13] devised a novel Bayesian formulation that combined dimensionality reduction, matrix factorization and binary classification for predicting drug-target interactions. The above supervised methods considered the unknown drug-target interactions as negative samples, which would largely influence the prediction accuracy. Xia et al. [14] proposed a semi-supervised learning method, NetLapRLS, to predict drug-protein interactions by using labeled and unlabeled information. Chen et al. [15] developed an inference method, NRWRH, by random walk on heterogeneous network, including protein-protein similarity network, drug-drug similarity network, and known drug-target interaction networks. Based on complex network theory, Cheng et al. [16] proposed a network-based inference method, NBI, for drug-target interaction prediction, which only utilized known drug-target interaction information. The common problem of the above three inference methods is that they cannot be applied to drugs without any known target information.

Taken together, the above mentioned methods for drug-target interaction prediction have various limitations and the difficulties of the prediction task lie in three aspects. Firstly, the known drug-target interactions are rare. Secondly, negative samples are hard or even impossible to select as there are no verified negative drug-target interactions. Thirdly, prediction should also be made to new drugs without any known target interaction information.

In this paper, a semi-supervised inference method NetCBP, utilizing both the small amount of available labeled data and the abundant unlabeled data together, has been proposed for drug-target interaction prediction based on the assumption that similar drugs often target similar proteins. We formulate the problem as a drug query problem. By querying the networks (the drug similarity network, the protein similarity network and the interaction network) with a given drug, a user expects to retrieve a list of target proteins with the highest predicted interactions with the given drug. The idea is that, if drugs are ranked by their relevance to the query drug, and proteins are ranked by their relevance to the hidden target proteins of the query drug, the known interactions between the most relevant drugs and proteins tend to be over-represented compared with random cases. We evaluated the method and existing methods with five-fold cross-validations in four classes of important drug–target interactions involving enzymes, ion channels, GPCRs and nuclear receptors. Experiments demonstrated that our method can achieve better performance. Furthermore, we discovered that some strongly predicted drug-target interactions were reported by publicly accessible databases. Finally, a comprehensive prediction of drug–target interactions was made using our method, which enables us to prioritize new potential drug–target interactions for drug development.

## Materials and Methods

### Data Preparation

In this study, four different drug–target interaction networks from humans, namely enzymes, ion channels, GPCRs and nuclear receptors, provided by Yamanishi et al. [10] are downloaded at http://web.kuicr.kyoto-u.ac.jp/supp/yoshi/drugtarget/. Here below we provide a brief description.

### Chemical Data

Chemical structures of drug compounds are extracted from the DRUG and COMPOUND sections in the KEGG LIGAND database [17]. Yamanishi et al. [10] calculate the structural similarities between drug compounds using SIMCOMP [18], which represents drug compounds as graphs and calculates a similarity score based on the size of the common substructures between two graphs. Given two drug compounds ***d_i_*** and ***d_k_***, chemical similarity between them is calculated based on the size of the common substructures between the two compounds using a graph alignment algorithm. The similarity matrix between all drug compound pairs is denoted as **D**.

### Genomic Data

Amino acid sequences of target proteins are extracted from the KEGG GENES database [17]. Yamanishi et al. [10] calculate the sequence similarities between target proteins using a normalized version of Smith–Waterman score [19]. Given two target proteins ***t_j_*** and ***t_l_***, genomic similarity between them can be found as 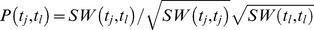, where **SW**(˙,˙) gives the canonical Smith–Waterman score and the similarity matrix between all target protein pairs is denoted as **P**.

### Drug-protein Interaction Data

At the time of the paper [10] was written, Yamanishi et al. [10] found 445, 210, 223, and 54 drugs targeting 664 enzymes, 204 ion channels, 95 GPCRs, and 26 nuclear receptors, receptively, and the known interactions are 2926, 1476, 635 and 90. The set of known drug–target interactions is regarded as ‘gold standard’ and is used to evaluate the performance of our proposed method in the cross-validation experiments as in the previous studies [10]–16.

### Method Description

We mainly consider the problem of predicting target proteins for a new drug without any known target interaction information.

### Problem Definition

We define the drug set as **Drug = {d_1_, d_2_, …,d_n_}** and the target protein set as **Protein = {p_1_, p_2_, …, p_m_}**, the drug-target interactions can be described as a bipartite DP graph **G(Drug, Protein, E)**, where **E = {e_ij_ : d_i_∈Drug, p_j_∈Protein}**. A link is drawn between **d_i_** and **p_j_** when the drug **d_i_** targets the protein **p_j_**. The DP bipartite network can be presented by an **n×m** adjacent matrix **{a_ij_}**, where **a_ij_ = 1** if **d_i_** and **p_j_** is linked, while all other unknown drug-target pairs are labeled as **0** to indicate they are going to be predicted. We define D (n*n), P (m*m), and a (n*m) as the adjacency matrix of the chemical structure similarity network, the sequence similarity network, and the drug-target interaction network, respectively. We query the networks with a drug to retrieve a target protein (or several proteins) predicted to interact with the query drug.

### Network-Consistency-based Prediction Method (NetCBP)

Under the assumption that similar drugs often target similar proteins, NetCBP integrates the chemical structure similarity data, the sequence similarity data and the drug-target interaction data. The idea of network consistency has been successfully used to predict gene-phenotype associations in [20]. The solid foundation for the algorithm can be traced back to [21]. Similar to [20], we formulate a graph query problem for drug and target protein interaction discovery. The query drug is represented by a binary vector **d = [d_1_, d_2_, …, d_n_]^T^** denoting the drug membership against the drug set, i.e. each **d_i_ = 1** if drug **i** is the query drug, otherwise **d_i_ = 0**. Similarly, the list of target protein is given by another binary vector **p = [p_1_, p_2_, …, p_m_]^T^** and protein **j** is a target protein if **p_j_ = 1**, otherwise **p_j_ = 0**.

To make full use of global network topological information, we compute the global relevance score between the query drug **d** and all the drugs based on the graph Laplacian of the drug structure similarity network D(n*n). We first normalize **D** as 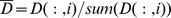, where *i* is the column number of **D**. A vector 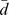 of graph Laplacian scores is derived from:
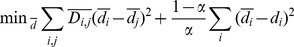
(1)


In Equation (1), the first term is a smoothness penalty, which forces connected drugs to receive similar scores, and the second term ensures the consistency with the query drug. Parameter**α**∈(0,1) balances the contributions from the two penalties. The close solution to Equation (1) is
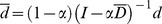
(2)


Similarly, the target sequence similarity network **P** is normalized as 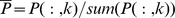, where *k* is the column number of **P**. Graph Laplacian scores can be derived to measure the relevance between the proteins and the target protein **p** with optimization of
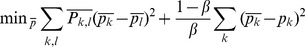
(3)with the close solution
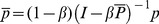
(4)where 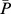 is the normalized P and parameter 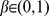.

Our method uses consistency in networks to measure whether the query drug **d** and a target protein **p** show coherent interaction with the known drug-target interactions. Specifically, given the graph Laplacian scores 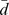, which ranks the drugs by their relevance to the query drug **d**, and the graph Laplacian scores 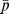, which ranks the proteins by their relevance to the hidden target protein **p**, NetCBP measures whether the interactions given by **a** are connecting drugs and proteins with similar scores in 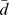 and 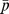. We simply go through each protein and compute a Pearson correlation coefficient score against the query drug d for each case.
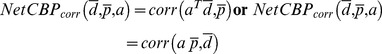
(5)


Finally, the protein(s) with the highest score(s) is chosen as the target protein(s). In Equation (4), there are two options and the one with a better prediction performance is selected.

## Results

In order to illustrate the effectiveness of our proposed method, we first compare NetCBP to other methods with five-fold cross-validation, and then present the results of two experimental scenarios: (i) predicting interactions for new drug compounds and (ii) predicting unknown interactions of the given network.

### Performance Evaluations and Comparison with Other Methods

To show the comparative performance of NetCBP in predicting interactions for new drugs, we perform five-fold cross-validation experiments on the four benchmark datasets for all methods. For each dataset, drug compounds are randomly split into five subsets of roughly equal size. Each subset is then used in turn as the test set and training is performed on the remaining four subsets. This procedure is repeated five times. This experimental procedure was also applied in [12] and [13]. We exactly follow the procedure in order to have comparable results.

Table 1 gives the average AUC (area under the receiver operating curve) values for DBSI [16], the method presented by Yanamishi *et al*. [12], KBMF2K [13] and our method NetCBP. The results produced by the best parameters (**α** = 0.2,**β** = 0.2) were reported in NetCBP. Compared with NBSI, our method receives higher average AUC values on all four datasets. Our method significantly improves the results on the class of nuclear receptors by ∼9%. It should be noted that the two methods TBSI and NBI presented in [16] cannot be applied to a new drug without known target interaction information.

**Table 1:** Prediction performances of DBSI [16], Yamanishi *et al.* (2010) [12], KBMF2K [13] and our method on the four benchmark datasets in terms of average AUC values.

Dataset	DBSI	Yamanishi*et al*. (2010)	KBMF2K	NetCBP
enzymes	0.8075	0.821	0.832	0.8251
ion channels	0.8029	0.692	0.799	0.8034
GPCRs	0.8022	0.811	0.857	0.8235
nuclear receptors	0.7578	0.814	0.824	0.8394

Compared with the supervised method presented by Yanamishi *et al*. [12] and the supervised method KBMF2K [13], our method achieves higher average AUC values on most the datasets. Our method improves the two supervised methods in another two aspects. One is that a huge number of samples will pose significant computational complexity to the two supervised methods [13]. Even though, KBMF2K shows improvements in time complexity, its time complexity is 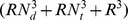
(**N_d_** and **N_t_** represent the numbers of drug compounds and target proteins. **R** Gives the dimensionality of the projected subspace.) [13]. Our method has lower time complexity and its time complexity is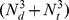. The other improvement is that our method does not use negative drug-protein interactions. Currently, experimentally verified negative drug-protein interactions are not available. Therefore, the use of these unconfirmed negative pairs may bring noise to the experiments.

In all, we can observe that NetCBP has obtained an excellent performance, which reveals that it can recovery verified drug-target interactions and hence has the potential to uncover potential drug-target interactions.

### Predicting Interactions for New Drug Compounds

In this experimental scenario, each drug in the four datasets was supposed to be a new drug. It was taken in turn as test dataset, and the remaining was used as the training dataset. We went through each protein and computed a Pearson correlation coefficient score against the ‘new’ drug. A high Pearson correlation coefficient score indicated a high possibility of a drug-target interaction. We rank the interaction pairs between a new drug and its target proteins with respect to their prediction scores. Take drug D00067 in the nuclear receptor dataset as an example. We consider the drug as a new drug and remove all its target interactions. The whole 26 potential targets are ranked according to our method. Two proteins-hsa:2099 (Estrogen receptor) and hsa:2100 (Estrogen receptor beta), both of which play crucial roles in many cancer types such as breast cancer [22] and prostate cancer [23].-are considered to be the most possible targets (rank 1 and rank 2, respectively) for the drug. We manually check and discover that the target hsa:2099 (Estrogen receptor) is in the benchmark datasets and the target hsa:2100 (Estrogen receptor beta) is confirmed by the database of KEGG [24]. The same things happen to drug D00312 and drug D00554 in the nuclear receptor dataset. The full lists of predicted ranks can be seen from Supplementary material (Material S1 for enzymes, Material S2 for ion channels, Material S3 for GPCRs and Material S4 for nuclear receptors).

When our method is applied to the benchmark dataset of enzymes, in about half of the predicted drugs (209 out of 445) the true solutions are contained within their top 1 scoring target proteins. In more than 60% of cases (274 out of 445) the true solutions are contained within their top 5 scoring target proteins. In more than 65% of cases (291 out of 445) the true solutions are contained within their top 10 scoring target proteins. Furthermore, we confirmed that 7 high-ranking (within top five, not reported in the benchmark datasets) interactions in the enzyme dataset (Table 2) are now annotated in at least one drug-target database, such as SuperTarget [1], KEGG [24], DrugBank [25] and ChEMBL [26].

**Table 2:** The newly confirmed drug-target interactions strongly predicted by NetCBP in the dataset of enzymes.

Drug ID	Target ID	Rank in the drug’s potential target proteins	Source
D00035	hsa:1636	1	SuperTarget
D00097	hsa:5743	2	ChEMBL, DrugBank
D00418	hsa:5742	1	DrugBank
D00448	hsa:5742	2	KEGG
D00542	hsa:1571	3	ChEMBL, DrugBank, KEGG
D00569	hsa:5742	3	DrugBank
D05458	hsa:4128	1	DrugBank

When our method is applied to the benchmark dataset of ion channels, in about a quarter of the predicted drugs (50 out of 210) the true solutions are contained within their top 1 scoring target proteins. In about 40% of cases (83 out of 210) the true solutions are contained within their top 5 scoring target proteins. In more than 54% of cases (114 out of 210) the true solutions are contained within their top 10 scoring target proteins. Furthermore, we confirmed that 13 high-ranking (within top five, not reported in the benchmark datasets) interactions in the ion channel dataset (Table 3) are now annotated in at least one of the above four drug-target databases [1], [24]–[26].

**Table 3:** The newly confirmed drug-target interactions strongly predicted by NetCBP in the dataset of ion channels.

**Drug ID**	**Target ID**	**Rank in the drug’s potential target proteins**	**Source**
D00110	hsa:6328	2	KEGG
D00252	hsa:6323	4	KEGG
D00303	hsa:6323	4	KEGG
D00438	hsa:779	3	KEGG
D00512	hsa:6323	5	KEGG, DrugBank
D00533	hsa:6328	2	KEGG
D00537	hsa:6331	1	KEGG
D00538	hsa:6331	1	KEGG, DrugBank
D00552	hsa:6331	1	KEGG
D00553	hsa:6328	2	KEGG
D00733	hsa:6328	2	KEGG
D05077	hsa:6328	2	KEGG
D06172	hsa:6328	2	KEGG

When our method is applied to the benchmark dataset of GPCRs, in more than 44% of the predicted drugs (99 out of 223) the true solutions are contained within their top 1 scoring target proteins. In 69% of cases (154 out of 223) the true solutions are contained within their top 5 scoring target proteins. In about 75% of cases (167 out of 223) the true solutions are contained within their top 10 scoring target proteins. Furthermore, we confirmed that 25 high-ranking (within top five, not reported in the benchmark datasets) interactions in the GPCR dataset (Table 4) are now annotated in at least one of the above four drug-target databases [1], [24]–[26].

**Table 4:** The newly confirmed drug-target interactions strongly predicted by NetCBP in the dataset of GPCRs.

Drug ID	Target ID	Rank in the drug’s potential target proteins	Source
D00079	hsa:5731	2	DrugBank
D00095	hsa:155	3	KEGG, SuperTarget
D00270	hsa:3358	5	KEGG
D00283	hsa:1814	4	ChEMBL, DrugBank
D00371	hsa:135	1	KEGG, DrugBank
D00415	hsa:3355	3	SuperTarget, DrugBank
D00419	hsa:5731	3	KEGG
D00442	hsa:6755	2	KEGG, DrugBank
D00498	hsa:4986	1	KEGG, DrugBank
D00604	hsa:148	4	ChEMBL
D00715	hsa:1129	2	KEGG
D00837	hsa:4985	4	DrugBank
D01103	hsa:1129	2	KEGG
D01386	hsa:153	1	KEGG
D01891	hsa:5732	2	KEGG
D02250	hsa:6751	1	KEGG
D02340	hsa:1812	3	DrugBank
D02349	hsa:154	1	KEGG
D02357	hsa:3358	2	KEGG, DrugBank
D02358	hsa:154	1	ChEMBL, DrugBank
D02725	hsa:5732	4	KEGG
D03490	hsa:155	3	KEGG
D04375	hsa:151	2	KEGG
D04625	hsa:154	1	KEGG
D05113	hsa:4986	1	DrugBank

When our method is applied to the benchmark dataset of nuclear receptors, in half of the predicted drugs (28 out of 54) the true solutions are contained within their top 1 scoring target proteins. In more than two-third of cases (37 out of 54) the true solutions are contained within their top 5 scoring target proteins. In more than 87% of cases (47 out of 54) the true solutions are contained within their top 10 scoring target proteins. Furthermore, we confirmed that 11 high-ranking (within top five, not reported in the benchmark datasets) interactions in the nuclear receptor dataset (Table 5) are now annotated in at least one of the above four drug-target databases [1], [24]–[26].

**Table 5:** The newly confirmed drug-target interactions strongly predicted by NetCBP in the dataset of nuclear receptors.

Drug ID	Target ID	Rank in the drug’s potential target proteins	Source
D00067	hsa:2100	2	KEGG
D00182	has:2099	2	ChEMBL
D00312	hsa:2100	2	KEGG
D00348	hsa:5915	3	ChEMBL
D00348	hsa:5916	5	ChEMBL
D00348	hsa:6258	4	ChEMBL
D00443	hsa:367	5	SuperTarget
D00554	has:2100	2	KEGG
D00690	has:2908	1	KEGG
D00898	has:2100	4	KEGG
D00962	hsa:2100	5	KEGG

### A Case Study

To illustrate the prediction performance of our method NetCBP on drugs, a case study about the drug clozapine (CLZ) was conducted. CLZ is considered one of the most effective therapeutic treatments for schizophrenia [27]. A clinical study demonstrated the necessity of moving CLZ from a 3rd line drug to a 1st line drug based on its overall benefit/risk ratio [27]. Therefore the identification of its targets could be of great importance.

We consider the drug as a new drug and its target interactions need to be predicted. The whole 664 potential targets in the class of enzymes are ranked according to our method. The five experimentally verified targets-hsa:1544 (Cytochrome P450 1A2), hsa:1557 (Cytochrome P450 2C19), hsa:1565 (Cytochrome P450 2D6), hsa:1576 (Cytochrome P450 3A4) and hsa:22954 (E3 ubiquitin-protein ligase TRIM32)-are ranked 25, 83, 4, 3, and 251 respectively, which means three out of the five targets are contained in the top 5% of the 664 potential targets. Meanwhile, we expect the prediction performance of our method could be improved by integrating more experimentally confirmed drug-target interactions.

### Comprehensive Prediction for the Given Network

After confirming the usefulness of our method, we conduct a comprehensive prediction of unknown interactions between all possible drugs and proteins on the four benchmark datasets. In the inference process for these predictions, we train NetCBP with all the known interactions. We rank the non-interacting pairs with respect to their interaction scores and extract the top 100 predicted interactions. The full lists of predicted interactions can be seen from Supplementary material (Material S5 for enzymes, Material S6 for ion channels, Material S7 for GPCRs and Material S8 for nuclear receptors).

We report the top three predicted interactions for each dataset. Table 6 lists the top three predicted interactions for each dataset. We manually check these predicted interactions from the latest online versions of SuperTarget [1], KEGG [24], DrugBank [25] and ChEMBL [26] databases. We confirm that 5 out of the 12 predictions are now annotated in at least one of these databases. We take these as strong evidence to support the practical application of our approach. Note that the predicted interactions that are not reported yet may also exist in reality.

**Table 6:** The top three predicted interactions on the four benchmark datasets.

Dataset	Drug ID	Target ID	Rank	Source	Dataset	Drug ID	Target ID	Rank	Source
enzymes	**D01441**	**hsa:1017**	**1**	**SuperTarget**	ion channels	D01768	hsa:6331	1	
	D00043	hsa:1990	2			**D06172**	**hsa:6328**	**2**	**KEGG**
	D01441	hsa:1018	3			**D00553**	**hsa:6328**	**3**	**KEGG**
GPCRs	**D03966**	**hsa:2914**	**1**	**SuperTarget**	nuclear receptors	D00094	hsa:3174	1	
	**D03966**	**hsa:2917**	**2**	**SuperTarget**		D00094	hsa:9971	2	
	D00283	hsa:886	3			D00094	hsa:6095	3	

## Discussion

In this manuscript, four important classes of drug-target interaction networks, including enzymes, ion channels, GPCRs and nuclear receptors, are studied. Compared with a small amount of experimentally verified drug-target interactions, there exist a large number of unknown drug-target interactions. Therefore, semi-supervised learning methods are very useful in addressing this problem of predicting target interactions for new drugs. Based on the foundations of previous research [20], [21], we presented a semi-supervised method named NetCBP for predicting drug-target interactions. Our method focuses on improving detection of drug-target interactions by integrating the drug similarity network and the target similarity network to better summarize sparse interactions for a global comparison of all possible drug-target interactions.

We use four benchmark datasets provided by Yamanishi et al. [10] to demonstrate the performance of our proposed method. Compared with DBSI [16], which uses only drug similarity information for drug-target interaction prediction, our method shows better prediction performance in all four benchmark datasets, especially in the class of nuclear receptors which has the fewest known drug-target interactions. It shows that integrating the drug similarity network and the target similarity network works better than only utilizing the drug similarity network in drug-target interaction prediction. Even compared with the two supervised learning methods presented in [12] and [13], our method shows superior prediction performance in most the classes of drugs. The two supervised learning methods [12], [13] have two drawbacks. Our method can overcome the two drawbacks. Meanwhile some strongly predicted drug-target interactions by our method are reported by the publicly available databases, which indicates the power of our method in realistic applications.

Despite the encouraging improvement, our method depends heavily on similarity values, Target similarity values received by Smith-Waterman scores heavily depend on the substitution matrix used [19]. From a technical viewpoint, the performance of our method could be improved by using more accurate similarity information designed for drugs and target proteins. Data incompleteness is another big issue for such prediction problem. Thus, the performance of our method could be further improved by integrating more verified drug-target interactions.

## Supporting Information

Material S1 **The ranks of interactions between each drug and its potential target proteins in the class of Enzyme.**
(XLSX)

Material S2 **The ranks of interactions between each drug and its potential target proteins in the class of Ion channel.**
(XLSX)

Material S3 **The ranks of interactions between each drug and its potential target proteins in the class of GPCR.**
(XLSX)

Material S4 **The ranks of interactions between each drug and its potential target proteins in the class of Nuclear receptor.**
(XLSX)

Material S5 **The predicted results with the top 100 highest scores in the class of Enzyme.**
(XLSX)

Material S6 **The predicted results with the top 100 highest scores in the class of Ion channel.**
(XLSX)

Material S7 **The predicted results with the top 100 highest scores in the class of GPCR.**
(XLSX)

Material S8 **The predicted results with the top 100 highest scores in the class of Nuclear receptor.**
(XLSX)
